# Tracking of an Oral *Salmonella*-Based Vaccine for Type 1 Diabetes in Non-obese Diabetic Mice

**DOI:** 10.3389/fimmu.2020.00712

**Published:** 2020-04-28

**Authors:** Jacques C. Mbongue, Ali Alhoshani, Jeffrey Rawson, Pablo A. Garcia, Nelson Gonzalez, Kevin Ferreri, Fouad Kandeel, Mohamed I. Husseiny

**Affiliations:** ^1^Department of Translational Research and Cellular Therapeutics, Diabetes and Metabolism Research Institute, Beckman Research Institute, City of Hope National Medical Center, Duarte, CA, United States; ^2^Department of Pharmacology and Toxicology, College of Pharmacy, King Saud University, Riyadh, Saudi Arabia; ^3^Faculty of Pharmacy, Zagazig University, Zagazig, Egypt

**Keywords:** *Salmonella*-based vaccine, sleeping beauty, transposons, Type 1 Diabetes, CD103^+^DC, TGFβ, tolerogenic dendritic cells

## Abstract

Type 1 diabetes (T1D) arises secondary to immune-driven destruction of pancreatic β-cells and manifests as insulin-deficient hyperglycemia. We showed that oral vaccination with live attenuated *Salmonella*, which simultaneously delivers autoantigens and a TGFβ expression vector to immune cells in the gut mucosa, provides protection against the progression of T1D in non-obese diabetic (NOD) mice. In this study we employed the Sleeping Beauty (SB) transposon system that is composed of a transposase and transposon encoding the td-Tomato to express red fluorescent protein (RFP) to permanently mark the cells that take up the *Salmonella* vaccine. After animal vaccination, the transposon labeled-dendritic cells (DCs) with red fluorescence appeared throughout the secondary lymphoid tissues. Furthermore, Sleeping Beauty containing *tgf*β*1* gene (SB-tgfβ1) co-expressed TGFβ and RFP. The labeled DCs were detected predominantly in Peyer’s patches (PP) and mesenteric lymph nodes (MLN) and expressed CD103 surface marker. CD103^+^ DCs induced tolerogenic effects and gut homing. TGFβ significantly increased programmed death-ligand-1 (PDL-1 or CD274) expression in the DCs in the MLN and PP of treated mice. Also, TGFβ increased cytotoxic T-lymphocyte-associated protein-4 (CTLA-4) levels in CD4^+^ cells in MLN and PP. Interestingly, DCs increased in all lymphatic organs of mice vaccinated with oral live *Salmonella*-based vaccine expressing preproinsulin (PPI), in combination with TGFβ, IL10, and subtherapeutic-doses of anti-CD3 mAb compared with vehicle-treated mice. These DCs are mostly tolerogenic in MLN and PP. Furthermore the DCs obtained from vaccine-treated but not vehicle-treated mice suppressed *in vitro* T cell proliferation. These data suggest that the MLN and the PP are a central hub for the beneficial anti-diabetic effects of an oral *Salmonella*-based vaccine prevention of diabetes in rodents.

## Introduction

Type 1 diabetes (T1D) results from auto-reactive killing of the pancreatic islet insulin-producing β-cells (1). Studies have shown that pre-proinsulin (PPI) is an initial autoantigen in non-obese diabetic (NOD) mice (2) and humans (3–5) promoting dysfunctional autoimmunity. Using oral antigen-specific vaccines of live attenuated (non-pathogenic) *Salmonella typhimurium* diabetic autoantigens have been delivered to mice (6, 7). Non-specific immune response in NOD mice was reported when an infection was induced with attenuated *S. typhimurium* (8–10). When orally administered, *Salmonella* moves from the gut to the gut-associated lymphoid tissues (GALT) of antigen presenting cells (APCs), resulting in formation of *Salmonella*-containing vacuoles (SCV) (11–13). *Salmonella* has two Type 3 Secretion Systems (T3SS) to invade and disseminate in organs (14). The T3SS encoded by *Salmonella* Pathogenicity Island 1 (SPI1-T3SS) is expressed by extracellular bacteria and is required for the invasion of non-phagocytic cells. The T3SS encoded by *Salmonella* Pathogenicity Island 2 (SPI2-T3SS) is expressed by intracellular *Salmonella* and is required for intracellular replication and systemic pathogenesis (15). The genes of SPI2-T3SS are induced inside the SCV and express specific antigens in GALT APCs. This has promoted the development of anti-cancer vaccines and a T1D vaccine (6, 10–12, 16–18). Fusion of heterologous antigens to specific SPI2-T3SS proteins causes them to be presented to lymphocytes within the gut mucosa (12, 19, 20). This minimizes and/or bypasses intestinal lumen antigen expression and subsequent degradation of the same. *Salmonella* can also deliver plasmids to host APCs and this feature has led to generation of DNA vaccines (21, 22). A novel diabetes vaccine enabled direct expression of tolerogenic cytokines like TGFβ and IL10 and induced tolerance to diabetic auto-antigens (10, 23). These APCs process and produce the antigens which migrate to other organs in the gut and stimulate the immune cells (9, 24).

Initial uptake of *Salmonella* vaccine by GALT-associated DCs helped promote oral tolerance (25). Antigens arising in the large bowel were transported to the GALT by migrating CD103^+^ DCs (25, 26). Tolerogenic dendritic cells (tolDCs) are especially active at suppressing immune activation (27–30). These effects are mediated through tolerogenic APC that induce T cell anergy, T cell apoptosis as well as induction of Tregs and type 1 regulatory T cells (Tr1) (28–35). CD103^+^ DCs induced tolerance and gut homing, protecting against colitis in mice. Also, reduction in celiac disease was attributed to the activity of CD11c^+^CD103^+^ DCs in rodents (36–38). We hypothesized that tolerogenic CD103^+^ DCs are intimately involved in the sampling and trafficking of the *Salmonella* antigen in NOD mice (37, 39–41). To identify the initial cell population mediating the oral vaccine effect, we developed a novel approach to label the cells that take up the *Salmonella* vaccine. It uses a transposon system known as Sleeping Beauty (SB;) (42) which is composed of Sleeping Beauty transposase and a transposon encoding the td-Tomato red fluorescent protein-expressing gene which permanently inserts into the genome of vertebrate animals (43, 44). Treating mice with *Salmonella* containing both plasmids enables passing of the plasmids to the host cells resulting in permanent marking of the cells. Using this approach permitted tracking and characterization of the cells mediating the vaccine effect. Here, we define the cells that uptake *Salmonella* and are essential for mediating the vaccine effect and characterize these cells as tolerogenic DCs based on the expression of regulatory molecules and secretion of cytokines.

## Materials and Methods

### Preparation of *Salmonella* Strain and Plasmid Construction

*Salmonella typhimurium* MvP728 (Δ*htrA/*Δ*purD*) has function as an oral vaccine vector (10, 23, 45). Bacteria were cultured in Luria-Bertani (LB) with or without chloramphenicol and/or carbenicillin (50 μg/ml).

The pSBbi-RP (#60513) (42) and pCMV(CAT)T7-SB100 (#34879) (46) plasmids for the Sleeping Beauty transposon system were from Addgene and kindly donated from the Kowarz and Izsvak Laboratories. The two plasmids were transformed into competent double mutant *S. typhimurium* MvP728 using electroporation (Bio Rad) after which sensitivity to carbenicillin and chloramphenicol was employed for selection.

**FIGURE 1:**
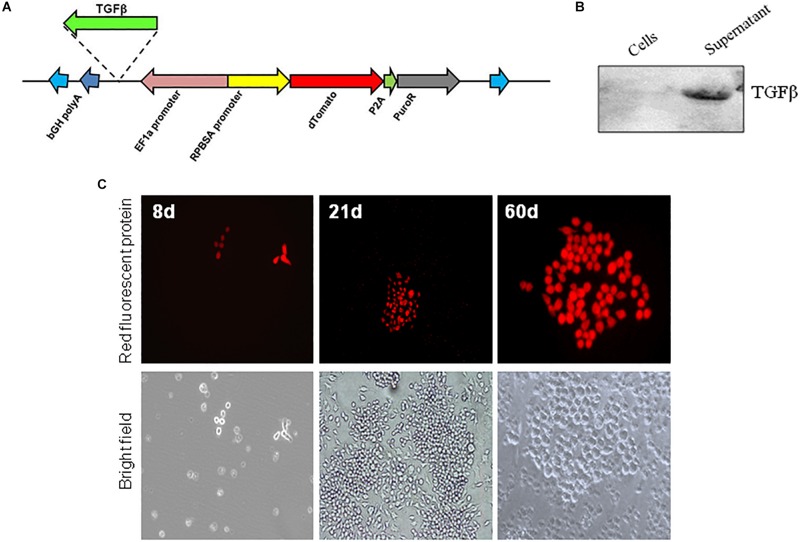
*In vitro* expression of Sleeping Beauty transposon in RAW 264.7 macrophages. **(A)** Plasmid (pSBbi-RP-TGFβ) construct for transposon coding for Td-Tomato and a puromycin resistance gene. The location for insertion of the mouse TGFβ on the plasmid is shown. *In vitro* infection of RAW 264.7 macrophages with *Salmonella*-carrying both the Sleeping Beauty Transposon plasmid pSBbi-RP or pSBbi-RP-TGFβ and transposase plasmid pCMV (CAT) T7 SB-100. **(B)** Western blot of cell lysate and supernatant performed 60 days after bacterial infection of macrophages using a DDK tag for TGFβ. **(C)** Expression of red fluorescent protein at 8, 21, and 60 days post-infection. Fluorescent images were acquired on a ZEISS inverted LSM700 microscope at 20x.

Mouse *tgfb1* sequence was amplified from pCMV6-Entry-*tgfb1* (Origene, MR227339) using forward primer 5′-CCATGGATGCCGCCCTCGGG-3′ and 5′-AAGCTTTTAAACCTTATCGTCGTCATCCTTG-3′ reverse primer. The PCR fragment was cloned using TOPO TA cloning kit (Thermo fisher, 450641) and confirmed by sequencing. Next, the *tgfb1* sequence was cut by *Nco*I/*Hin*dIII then subcloned into *Nco*I/*Hin*dIII digested pSBbi-RP plasmid to yield pSBbi-RP-TGFβ (Figure 1A). The pSBbi-RP-TGFβ and pCMV(CAT)T7-SB100 plasmids were transformed into *Salmonella* as described (47).

### *In vitro* Infection

The murine RAW264.7 macrophage cell line was obtained from the American Type Culture Collection (ATCC no.-TIB-71). The cells were plated in 24-well plates (5 × 10^4^ cells per well) and were allowed to attach overnight at 37°C in an atmosphere of 5% CO_2_. Overnight cultures of *Salmonella* containing pCMV(CAT)T7-SB100 and pSBbi-RP or pSBbi-RP-TGFβ were diluted and added to the cells growing in 24 well tissue culture plates at a multiplicity of infection of about 20. The bacteria were centrifuged onto the cells at 500 × *g* for 5 min. The plates were placed in an incubator for 25 min at 37°C for uptake of bacteria. Wells were washed three times with PBS and then incubated for 1 h in media containing 100 μg/ml of gentamicin to kill extracellular bacteria. Subsequent growth media were supplemented with 10 μg/ml of gentamicin for the remainder of the experiment. Given that pSBbi-RP confers resistance to puromycin, RAW 264.7 macrophages that incorporated the transposon were positively selected by the addition of puromycin (2 μg/ml). Cultures were treated with *Salmonella*-expressing both pSBbi-RP or pSBbi-RP-TGFβ and pCMV(CAT)T7-SB100 and cultured from 7 to 60 days for expression analysis. A ZEISS inverted LSM700 microscope was employed to obtain images that were processed with ZEN-lite digital imaging software (CarlZeiss, Oberkochen, Germany) for processing. The expression of TGFβ by macrophages was analyzed in cell lysate or culture supernatant by Western blot analysis.

### Animal Experiments

Seven-week-old female NOD/ShiLtJ mice (Jackson Laboratory, Bar Harbor, ME, United States) were housed in pathogen-free conditions. City of Hope has been fully accredited by the Association for Assessment and Accreditation of Laboratory Animal Care International (AAALAC). The study was approved by the Institutional Animal Care and Use Committee of City of Hope (IACUC# 11032).

Eight-week-old NOD mice were vaccinated on days 0 and 7 by oral gavage with 10^7^CFU/mouse in a total volume of 200 μl of 5% sodium bicarbonate as described (10, 23, 45). Furthermore, vaccinated animals were treated with hamster anti-CD3 mAb for five consecutive days (days 0–4; 2.5 μg i.p./mouse).

### Flow Cytometry

Single cell suspensions of spleen, pancreatic lymph nodes (PLN), mesenteric lymph nodes (MLN), and Peyer’s patches (PP) were digested in collagenase D (1 mg/ml) at week 4 post-vaccination. Cells were stained directly with the following conjugated antibodies: MHC-II FITC anti-mouse I-Ak (Aβk), APC anti-mouse CD11c, Brilliant Violet 605 anti-mouse CD103, PE anti-mouse CD80, Brilliant Violet 711 anti-mouse CD86 and matching isotype controls. Cells were also stained with anti-mouse CD11b PE/Cy7, BV650 anti-mouse/human CD45R/B220, BV510 anti- mouse Ly-6G/Ly-6C (Gr-1) and matching isotype controls. Signal height and width were used to exclude doublets. Dead cells were excluded using Fixable Live/Dead Yellow Stain (Invitrogen), according to the manufacturer’s protocol. All antibodies and isotypes were obtained from Biolegend (San Diego, United States). A BD Fortessa flow cytometer (BD Biosciences) and FlowJo software gated on isotype controlled single DAPI-negative cell populations was used for FACS.

### *In vitro* Proliferation Assay and Cytokine Assay

Responder T cells (Tresp) were generated and isolated from spleens of NOD mice (3 mice) immunized with insulin peptide B9-23 (100 μg) and emulsified in complete Freund adjuvant (CFA). After 11 days CD4^+^CD25^–^ Tresp cells were isolated from pooled splenocytes using negative selection for CD4^+^ followed by negative selection for CD25^+^ using a CD4^+^CD25^+^ regulatory T Cell Isolation Kit (Miltenyi Biotec, 130-091-041) and then labeled with 1:1000 of 5 mM Cell Trace Violet (CTV) (48). CD4^+^CD25^+^ Treg cells were isolated from pooled splenocytes of non-vaccinated NOD mice (5 mice) using negative selection for CD4^+^ followed by positive selection for CD25^+^ using a CD4^+^CD25^+^ regulatory T Cell Isolation Kit (Miltenyi Biotec, 130-091-041). Dendritic cells (DCs) were isolated from pooled splenocytes (5 mice) isolated from either vaccinated or non-vaccinated mice using PE-conjugated CD11c antibody (Miltenyi, 130-110-701) followed by anti-PE microbeads (Miltenyi Biotec, 130-091-041). The Tresp cells (1 × 10^5^ cells/well) were co-cultured in 96-well round bottom plates with or without 1 × 10^5^ Treg cells isolated from non-vaccinated mice using different ratios of DCs isolated from either vaccinated or non-vaccinated mice in the presence of insulin peptide B9-23 (10 μg/ml). After 3 days, the proliferation of CD4 responder T cells positive for CTV and with surface expression of CD44 and CD69 (signifying cell activation) was analyzed by gating on proliferated cells using FlowJo software 10.4.

Cell free supernatants were collected for detection of IL10, TGFβ, IFNγ, and TNFα using Mouse Quantikine ELISA Kits (R&D Systems) according to the manufacturer’s instructions.

### Gene Expression

RNA isolated from sorted CD11c^+^ and CD3^+^CD4^+^ cells obtained from splenocytes, PLN, MLN, and PP were isolated using the DirectZol kit (Zymo Research). Between 3 and 5 × 10^5^ cells were sorted with 80 to 90% viability. RNA concentration was measured with a Nanodrop 2000 (Thermo Scientific). A 260/280 ratio of 1.8 to 2.00 was considered for RNA purity. Total RNA (500 ng) was used for cDNA synthesis using the qScript cDNA SuperMix kit (Quantabio). qPCR analysis was performed using TaqMan gene expression assays (Applied Biosystem) according to the manufacturer’s protocol. qRT-PCR was done with ABI Fast 7500 software (Applied Biosystems, ABI). Primers and probes were purchased from Applied Biosystems: CD274 (Mm03048248_m1), and CTLA4 (Mm00486849_m1). Relative changes in mRNA were calculated using the ΔΔCt method. Ct values of target genes were calculated relative to a reference control gene (TATA box binding protein, Tbp (Mm01277042_m1) using the following formula ΔCt = Ct_*Target*_-Ct_*Reference*_. Expression level = 2^–^^Δ^ ^*Ct*^.

### Statistical Analyses

Two-way ANOVA were used for analysis of the percentage of positive cells between groups and to compare cell populations after FACS analysis. A *p* < 0.05 was considered significant. Statistical analysis was performed using GraphPad Prism 8 software.

## Results

### *In vitro* Expression of Sleeping Beauty

RAW 264.7 macrophages were infected *in vitro* with *S. typhimurium* carrying both Sleeping Beauty Td-Tomato transposon plasmid (pSBbi-RP) and the transposase plasmid pCMV(CAT)T7-SB100 (SB100) (Figure 1A). The expression of TGFβ was detected in supernatant only after infection of macrophages with pSBbi-RP-TGFβ and pCMV(CAT)T7-SB100 (Figure 1B), implying that TGFβ was secreted after expression by host cells under the control of the CMV promoter.

Additionally the expression of RFP after infection of RAW macrophages was detected. RAW macrophages infected with the pSBbi-RP transposon, but not the pCMV(CAT)T7-SB100 transposase plasmid, were negatively selected by the addition of 2 μg/ml of puromycin. pCMV(CAT)T7-SB100 which codes for transposase conferred long-term resistance to puromycin in RAW macrophages as well as permanent expression of RFP. The presence of red colonies was seen consistently at 7–8 days post-infection (Figure 1C). The expression of the RFP appeared to be permanent as the fluorescent colonies grown for more than 60 days post-infection continued to show signal (Figure 1C).

### *In vivo* Labeling of Antigen-Presenting Cells

**FIGURE 2:**
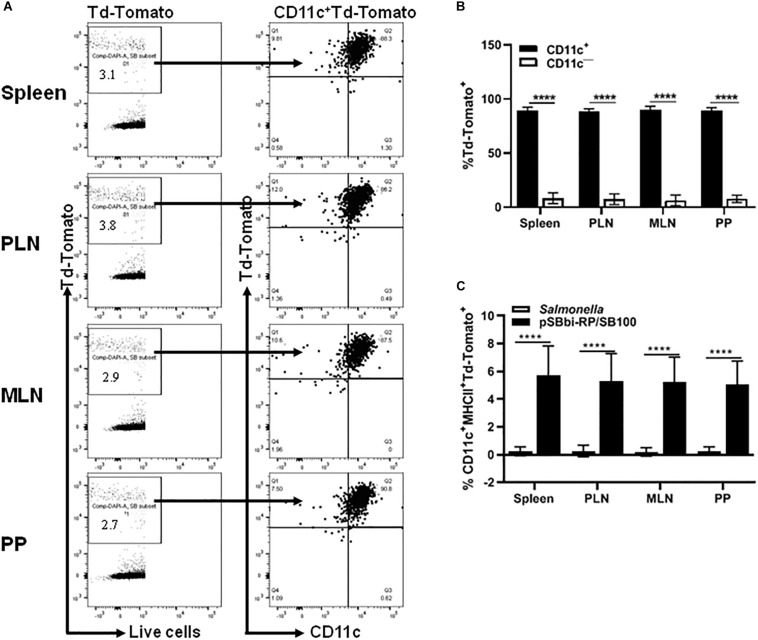
*In vivo* detection of the red fluorescent protein in different organs of vaccinated NOD mice. NOD mice were given a single oral dose of *Salmonella*-containing plasmid (pSBbi-RP/SB100, *n* = 10) compared to *Salmonella* only (*n* = 10). Four weeks post-vaccination, red fluorescent protein was detected in different organs. **(A)** Representative FACS plots gated the frequency of live red-labeled CD11c^+^ and CD11c^–^ cells in the spleens, pancreatic lymph nodes (PLN), mesenteric lymph nodes (MLN), and Peyer’s patches (PP) of mice from each group. **(B)** Quantification of the percentages of the CD11c^+^ and CD11c^–^ red-labeled cells in all organs. **(C)** Quantification of the percentages of red-labeled CD11c^+^MHCII^+^ in total live cells isolated from the spleen, PLN, MLN, and PP. The data shown are as the average with standard deviation (SD) obtained from two independent experiments. Statistical analysis using two-way ANOVA shows significance between groups (****p < 0.0001).

Although *Salmonella*-mediated labeling of RAW cells occurred *in vitro*, it was not known whether this cell labeling would occur *in vivo*. Mice were administered a single dose of *Salmonella*-containing both transposon (pSBbi-RP) and transposase (SB100) plasmids. Four weeks later, flow cytometry demonstrated red fluorescent cells in the PP, MLN, PLN, and spleen of inoculated NOD mice. Characterization showed that the majority of red-labeled cells were CD11c^+^ indicating that they were indeed DCs (Figure 2A). However, a minority of red-labeled cells were found to be other than DCs (Figure 2B). Further characterization of red-labeled DCs showed that they were MHCII^+^ APCs (Figure 2C). Overall, red-labeled MHCII^+^CD11c^+^ DCs comprised 5 to 10% of total live cells in the secondary lymphoid tissues (Figure 2C).

### TGFβ Induces Higher Levels of DCs

**FIGURE 3:**
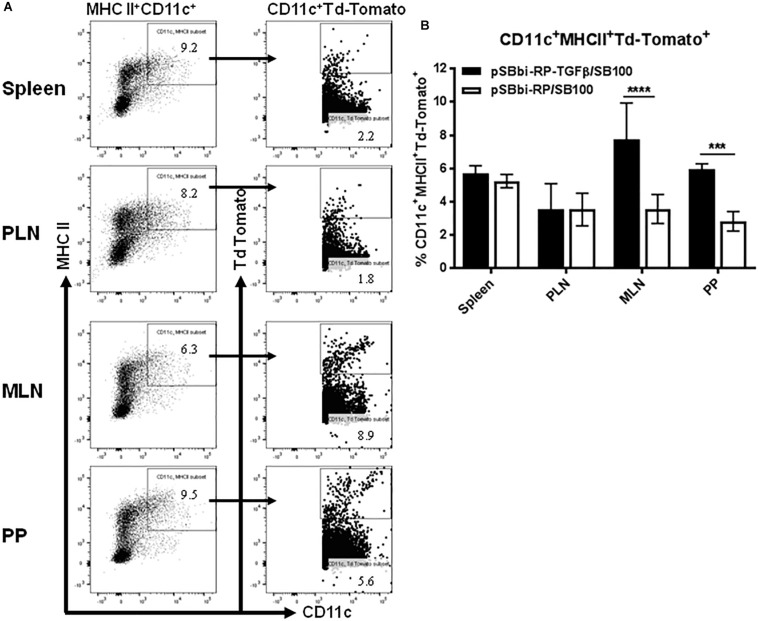
Vaccine-delivered TGFβ alters levels of DCs in secondary lymphoid tissues. NOD mice were given a single oral dose of *Salmonella* (pSBbi-RP-TGFβ/SB100, *n* = 10) or *Salmonella* (pSBbi-RP/SB100, *n* = 10). Four weeks post-vaccination, red fluorescent protein was detected in DCs of different organs. **(A)** Representative FACS plots gated the frequency of MHCII^+^CD11c^+^Td-Tomato^+^ cells in the spleens, PLN, MLN, and PP of mice from each group. **(B)** Quantification of the percentages of MHCII^+^CD11c^+^Td-Tomato^+^ cells in the spleen, PLN, MLN, and PP. The data shown are the average with SD from two independent experiments. Statistical analysis using two-way ANOVA shows significance between *Salmonella* (pSBbi-RP-TGFβ/SB100) and *Salmonella* (pSBbi-RP/SB100) groups (***p < 0.001, ****p < 0 0001).

An important component of the T1D *Salmonella*-based vaccine is the combination of diabetic autoantigens with TGFβ. TGFβ is used as an immunomodulator and is expressed by the host cells under the CMV promoter. To investigate the effect of TGFβ on the red-labeled cell populations, the mouse *tgf*β*1*-coding region was inserted into the pSBbi-RP transposon plasmid upstream of the EF1a promoter to create pSBbi-RP-TGFβ (Figure 1A) that express both TFGβ and RFP. Mice were given the *Salmonella-*containing both pSBbi-RP-TGFβ and transposase (SB100) orally and compared with animals receiving the *Salmonella*-containing the pSBbi-RP/SB100 plasmids. Incorporation of the pSBbi-RP-TGFβ transposon into the host cell genome conferred both red fluorescent labeling and host cell secretion of mouse TGFβ. Four weeks after administration of *Salmonella*, labeled cells were found to be red fluorescent MHCII^+^CD11c^+^ DCs which subsequently migrated to the spleen, MLN, PLN, and PP (Figures 3A,B). However, in this situation the presence of TGFβ appeared to induce a significant increase in the number of red fluorescent MHCII^+^CD11c^+^ DCs in the PP and MLN (two-way ANOVA, *p* < 0.001, *p* < 0.0001, Figure 3B) compared to mice given *Salmonella* lacking the mouse *tgf*β*1*-coding region.

### TGFβ-Induces Gut CD103^+^ DCs

**FIGURE 4:**
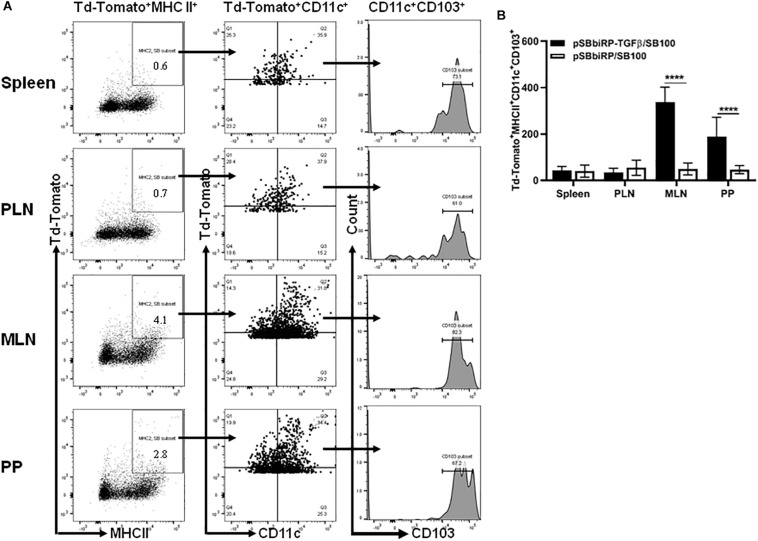
Vaccine-delivered TGFβ alters levels of gut DCs. NOD mice were given a single oral dose of *Salmonella* (pSBbi-RP-TGFβ/SB100, *n* = 10) or *Salmonella* (pSBbi-RP/SB100, *n* = 10). Four weeks post-vaccination, red fluorescent protein was detected in DCs of different organs. **(A)** Representative FACS plots gated the frequency of the live red-labeled MHCII^+^CD11c^+^CD103^+^ cells in the spleens, PLN, MLN, and PP of mice from each group. **(B)** Quantification of the numbers of MHCII^+^CD11c^+^CD103^+^ in red-labeled cells in the spleen, PLN, MLN, and PP. The data shown are the average with SD from two independent experiments. Statistical analysis using two-way ANOVA shows significance between *Salmonella* (pSBbi-RP-TGFβ/SB100) and *Salmonella* (pSBbi-RP/SB100) groups (****p < 0.0001).

The MHCII^+^CD11c^+^CD103^+^ populations in mice administered *Salmonella*-containing pSBbi-RP-TGFβ/SB100 plasmids with and without TGFβ were assessed 4 weeks after inoculation (Figure 4A). Interestingly, very few red fluorescent DCs from mice given vaccine lacking TGFβ were CD103^+^. However, mice given vaccine with TGFβ showed a significant increase in PP and MLN of red-labeled DCs that were also CD103^+^ (two-way ANOVA, *p* < 0.0001, Figure 4B).

### *Salmonella*-Delivered TGFβ Increases PDL-1 and CTLA-4 Expression Levels in Secondary Lymphoid Tissues

**FIGURE 5:**
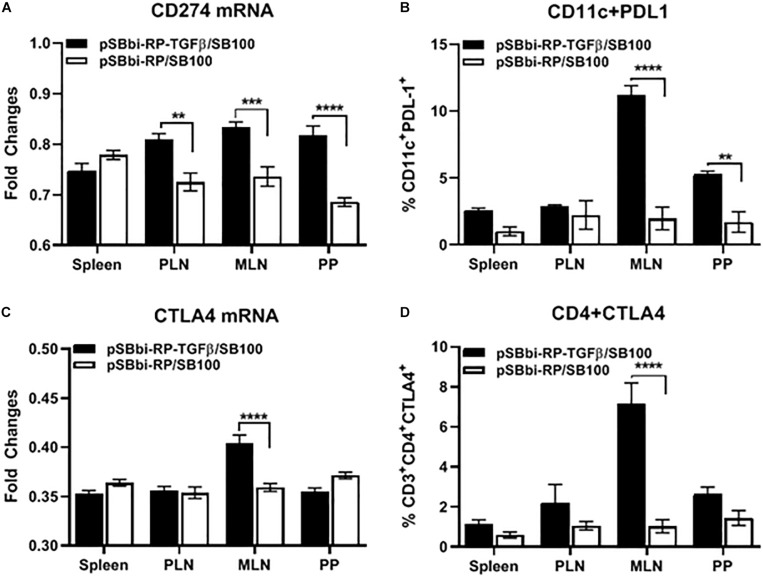
Endogenous TGFβ alters PDL-1 and CTLA4 expression in secondary lymphoid tissues. NOD mice were given a single oral dose of *Salmonella* (pSBbi-RP-TGFβ/SB100) or *Salmonella* (pSBbi-RP/SB100). Four weeks post-vaccination, the fold changes in gene expression of **(A)** CD274 (PDL-1) in sorted CD11c cells and **(C)** CTLA4 in sorted CD4 cells isolated from specified organs was determined. Percentage of CD11c^+^ cells expressing PDL-1 **(B)** or CD4^+^ cells expressing CTLA4 **(D)** were quantified in the spleen, PLN, MLN, and PP. The data shown are the average with SD from two independent experiments. Statistical analysis using two-way ANOVA shows significance between *Salmonella* (pSBbi-RP-TGFβ/SB100) and *Salmonella* (pSBbi-RP/SB100) groups (**p < 0.01, ***p < 0.001, ****p < 0 0001).

Cells from animals given *Salmonella*-containing pSBbi-RP-TGFβ/SB100 plasmids with and without TGFβ were compared. The CD11c^+^ DCs and CD4^+^ T-cells from splenocytes, MLN, PLN and PP were sorted for qRT-PCR analysis. Programmed death-ligand-1 (PDL-1or CD274) expression was significantly increased in DCs in PLN, MLN, and PP but not the spleen of NOD mice treated with TGFβ-containing plasmid (two-way ANOVA, *p* = 0.001, *p* = 0.0003, *p* > 0.0001) compared with the plasmid without TGFβ (Figure 5A). Furthermore the percentage of DCs expressing PDL-1 measured by flow cytometry was significantly elevated in MLN and PP but not spleen and PLN of mice treated with TGFβ-containing plasmid (two-way ANOVA, *p* < 0.0001, *p* = 0.002) compared with the plasmid without TGFβ (Figure 5B). Interestingly, cytotoxic T-lymphocyte-associated protein-4 (CTLA-4) expression was significantly increased in CD4^+^ T-cells in MLN of mice treated with TGFβ-containing plasmid (two-way ANOVA, *p* < 0.0001) compared with the plasmid without TGFβ (Figure 5C). FACS also showed that CTLA-4 is significantly elevated on the surface of CD4^+^ T cells in the MLN mice treated with TGFβ-containing plasmid (two-way ANOVA, *p* < 0.0001) compared with the plasmid without TGFβ (Figure 5D).

### A *Salmonella*-Based Vaccine Increases the DCs in Lymphoid Organs

**FIGURE 6:**
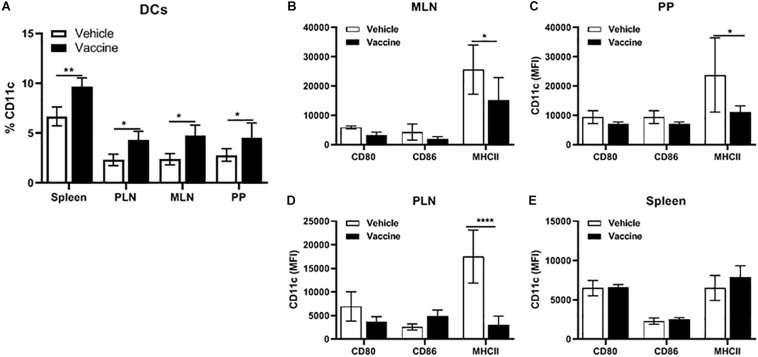
A *Salmonella*-based vaccine increase DCs in lymphoid organs. NOD mice were injected with 2.5 μg/mouse of anti-CD3 mAb for 5 days followed by 2 doses of oral vaccine containing PPI + TGFβ + IL10 (*n* = 5). Four weeks post-vaccination, DCs were analyzed in different organs by FACS analysis. **(A)** Quantification of the percentage of CD11c^+^ in total live cells of the spleen, PLN, MLN, and PP. **(B)** MLN, **(C)** PP, **(D)** PLN, and **(E)** spleen expression of CD80, CD86, and MHCII was measured in DCs from each organ. The data shown are the average with SD from two independent experiments. Statistical analysis using two-way ANOVA shows significance between vaccine and vehicle treated groups (*p < 0.05, **p < 0.01, ****p < 0.0001).

Pre-diabetic NOD mice were vaccinated with *Salmonella*-based vaccine (PPI + TGFβ + IL10 + anti-CD3) to quantify the percentage of DCs in the spleen, PLN, MLN, and PP. Figure 6A shows the frequency of CD11c^+^ DCs was 5 to 10% of the total number of live cells in all detected lymphoid organs. The percentage of DCs was significantly increased in all organs isolated from vaccine-treated mice compared to vehicle (two-way ANOVA, *p* < 0.01, *p* < 0.05). Additionally, we could not detect any differences between vaccine or vehicle treated mice in the percentage of cell populations that were characterized as B220^+^GR-1^–^ (B cells, Supplementary Figure S1A) and CD11b^+^GR-1^+^ as polymorph nuclear macrophages (PMN, Supplementary Figure S1B) in the spleens, PLN, MLN, and PP.

Moreover, CD11c^+^ DCs from vaccinated mice showed lowered expression of the MHCII molecule and co-stimulatory molecules CD80, and CD86 in cells from MLN (Figure 6B), PP (Figure 6C) and PLN (Figure 6D) compared to DCs from these lymphoid organs from vehicle-treated mice. In contrast, CD11c^+^ DCs from the spleen from vaccinated mice (Figure 6E) showed no difference in the level of expression of MHCII, CD80 and CD86 compared to DCs from vehicle-treated mice.

### Importance of DCs for *Salmonella*-Based Vaccine Therapy

**FIGURE 7:**
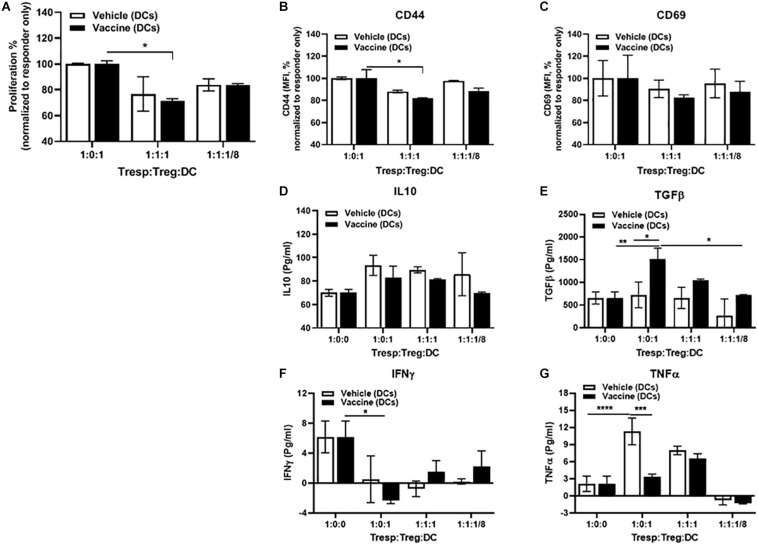
DCs mediated *in vitro Salmonella*-based anti-inflammatory effects. Female 8-week-old NOD mice were treated with 2.5 μg/mouse of anti-CD3 mAb via i.p. injection for 5 days followed by 2 doses of oral combined vaccines. Splenocytes were harvested from the indicated group at day 30 post-vaccination. CD4^+^CD25^–^ Tresp cells isolated from splenocytes of B9-23 vaccinated mice were dye-labeled, co-cultured with CD4^+^CD25^+^ Treg cells isolated from non-vaccinated mice, and DCs from vaccinated or non-vaccinated mice for 3 days with insulin peptide B9-23 (10 μg/ml). **(A)** Proliferation of Tresp was measured by flow cytometry of dye dilution and shown as the percentage of Tresp normalized to Tresp only culture. **(B)** Activation of Tresps, shown as MFI of CD44 expression or **(C)** CD69 normalized to Tresp-only culture. ELISA for regulatory **(D)** IL-10, and **(E)** TGFβ. ELISA for inflammatory **(F)** IFNγ, and **(G)** TNFα. The data shown are the average with SD from two independent experiments. Statistical analysis was performed using two-way ANOVA (*p < 0.05, **p < 0.01, ***p < 0.001, ****p < 0.0001).

We next evaluated whether the DCs in vaccine-treated NOD mice were tolerogenic. As responder T cells, we used CD4^+^CD25^–^ T cells isolated from spleens of normoglycemic B9-23 vaccinated NOD mice. We found that CD4^+^CD25^+^ Treg cells from vehicle-treated mice efficiently suppressed proliferation and activation of responder T cells when combined with equal amount of DCs from vaccinated mice but did not alter proliferation when cultured with DCs from vehicle-treated animals (Figure 7A). As well, these tolerogenic DCs lowered expression of activation markers CD44 (Figure 7B) and CD69 (Figure 7C). Additionally DCs from vaccine-treated mice, when combined with Tregs, efficiently increased regulatory cytokines such as IL10 (Figure 7D) and TGFβ (Figure 7E) and decreased the inflammatory cytokines such as IFNγ (Figure 7F) and TNFα (Figure 7G). In contrast DCs from vehicle-treated mice, when combined with Tregs decreased the regulatory cytokines IL10 (Figure 7D) and TGFβ (Figure 7E) and increased the inflammatory cytokines IFNγ (Figure 7F) and TNFα (Figure 7G). Finally, when we decreased the number of DCs to a ratio of 1:8, the suppressor activity of Tregs decreased indicating that the DCs from vaccinated animals were important to the suppressor activity of the Tregs.

## Discussion

Recently we developed an oral vaccine using live attenuated *Salmonella* that prevented and reverted the onset T1D in NOD mice (45). This vaccine was a combination of PPI + TGFβ + IL10 and anti-CD3 mAb. We showed that the vaccine increased Tregs and Tr1 cells in vaccinated mice (23, 45). To understand how *Salmonella* prevented diabetes, it was important to first identify the cell populations that interact with the microorganism and to investigate the role of *Salmonella*-delivered TGFβ in creation of a tolerogenic micro-environment in NOD mice. On oral administration, *Salmonella* gains access to the individual via DCs that phagocytosis the bacteria, through SPI1-T3SS-mediated invasion of other cells or by GALT cells. Indeed, these several mechanisms likely contribute to wider spread of the bacteria beyond the gut. In specialized lymphatic organs such as PP and MLN, the bacteria is mostly intracellular, its survival and replication dependent on the function of SPI2-T3SS (49).

Transposable elements are naturally occurring vehicles and are used for transgenesis and mutagenesis. The Sleeping Beauty transposon shows efficient transposition in a wide range of vertebrate cells, including humans (50, 51, 52). The current study employed attenuated *Salmonella* as a vehicle to deliver the transposon plasmid expressing a detectable fluorescent protein to host cells and allowed tracking of *Salmonella*-bearing host cells throughout the GALT. Secondary lymphoid organs provide the specialized microenvironment necessary for generating an immune-specific response. DCs, while present in low numbers, are distributed widely and beyond lymphatic tissues. Because DCs work very efficiently at controlling T cells, they are critical to turning on or off immune responses (53). We found that the DCs labeled after oral administration of *Salmonella* were present in all lymphoid tissues examined, consistent with results from others.

Previous studies have shown that tolerance to oral antigens is mediated by CD103^+^ DCs in the lamina propria (39, 41, 54–56). CD103^+^ DCs are unique to the gut. Transforming growth factor receptor 1 (TGFβR1) has a cell-intrinsic role in the development of these cells. Together, these published data suggested that MHCII^+^CD11c^+^CD103^+^ cells mediate the regulatory effects of the *Salmonella*-based vaccine. In keeping with this, TGFβ promoted the upregulation of DC cell surface CD103 in animals administered the vaccine. In the intestinal tract, CD103^+^ DCs carry acquired antigens to the local lymph nodes where they encounter CD8^+^ T cells (57). By promoting naïve T cell transformation to regulatory Foxp3^+^ T cells, DCs promote immune suppression (39, 54, 56). Our results demonstrate that *Salmonella*-delivered TGFβ recruits CD103^+^ DCs in the MLN and PP. These data suggest that *Salmonella*-autoantigen driven education of the naïve T-cells into iTregs or Tr1 occurs specifically in these secondary lymph nodes (45). Our data further indicate that TGFβ is involved in the trafficking and control of CD103^+^ DCs, concentrating their function within MLN and PP.

Additionally, it appears that TGFβ has an impact on PDL-1 and CTLA-4 expression in NOD mice. PD-L1 suppresses the immune system in cancer and autoimmune disease (58). Endogenous ligand-receptor pathways, such as the PD-L1/PD-1, limit T cell antigen activation and promote regulatory T cell survival (58, 59). Similarly, CTLA-4 limits T cell activation in part by blunting CD28 co-activation (60). Indeed, some data suggests that variation in the CTLA-4 gene may promote autoimmunity (61–64). Our data suggest that TGFβ increases PDL-1 and CTLA-4 at the lymphoid sites where CD103^+^ DCs are found to be elevated consistent with a tolerogenic microenvironment. As some time differentiation of naïve T cells into nTregs or Tr1 occurs in the MLN and the PP (31, 34, 54, 56, 65) and these lymphoid sites appear to be the central hub for *Salmonella* vaccine activity.

Some studies characterized semi-mature phenotype DCs that are thought to be most effective in inducing tolerance in autoimmune diseases (66, 67). In this study, our combined therapy increased DCs in all lymphatic tissues of vaccinated mice. Additionally, these DCs expressed low levels of co-stimulatory molecules. Moreover, *in vitro* proliferation assays conformed that the DCs from vaccinated mice were essential for suppressor activity of Tregs by decreasing proliferation and activation of effector T cells. Furthermore, these DCs secreted less inflammatory cytokines such as TNFα, and IFNγ while secreting increased tolerogenic cytokines such as IL10 and TGFβ.

Together, the data herein suggest the oral vaccine could be an effective immunotherapy to reestablish tolerance to self-antigens and of value to individuals with T1D. The vaccine may also find use in the treatment of other autoimmune disorders. Using *Salmonella* offers several advantages over other methods of drug delivery. First, oral delivery exploits the natural tolerogenic functions of the GALT system. Second, oral delivery is a patient-friendly treatment approach. Third, inclusion of TGFβ and possibly other tolerogenic cytokines should improve the efficacy of the vaccine. Fourth, protection of the antigen from degradation while it traverses the digestion system is important. Lastly for clinical relevance, a vaccine for typhoid fever that uses the *Salmonella typhi* Ty21a strain is approved by the FDA.

## Data Availability Statement

The datasets generated for this study are available on request to the corresponding author.

## Ethics Statement

The animal care facility at City of Hope has been fully accredited by the Association for Assessment and Accreditation of Laboratory Animal Care International (AAALAC). The study was approved by the Institutional Animal Care and Use Committee (IACUC# 11032).

## Author Contributions

JM, KF, and MH conceived and designed the experiments. JM, AA, JR, PG, NG, and MH performed the experiments. KF and MH analyzed the data. JM, FK, and MH wrote the manuscript.

## Conflict of Interest

The authors declare that the research was conducted in the absence of any commercial or financial relationships that could be construed as a potential conflict of interest.
