# Supplementary material for: Tracking of an Oral Salmonella-Based Vaccine for Type 1 Diabetes in Non-obese Diabetic Mice
Source: Front Immunol. 2020 Apr 28;11:712. doi: 10.3389/fimmu.2020.00712 (PMC7198770; doi:10.3389/fimmu.2020.00712)
Supplement: Supplementary file 1 [file Data_Sheet_1.PDF]

Tracking of an oral *Salmonella*-based vaccine for type 1 diabetes in non-obese diabetic mice

Jacques C. Mbongue [1], Ali Alhoshani [2], Jeffery Rawson [1], Pablo A. Garcia [1], Nelson Gonzalez [1], Kevin Ferreri [1], Fouad Kandeel [1], and Mohamed I. Husseiny [1,3,*]

[1]Department of Translational Research & Cellular Therapeutics, Diabetes & Metabolism Research, Institute, Beckman Research Institute of City of Hope National Medical Center, Duarte, California 91010-3000, USA
[2]Department of Pharmacology and Toxicology, College of Pharmacy, King Saud University, Riyadh, Saudi Arabia
[3]Faculty of Pharmacy, Zagazig University, Zagazig, Egypt

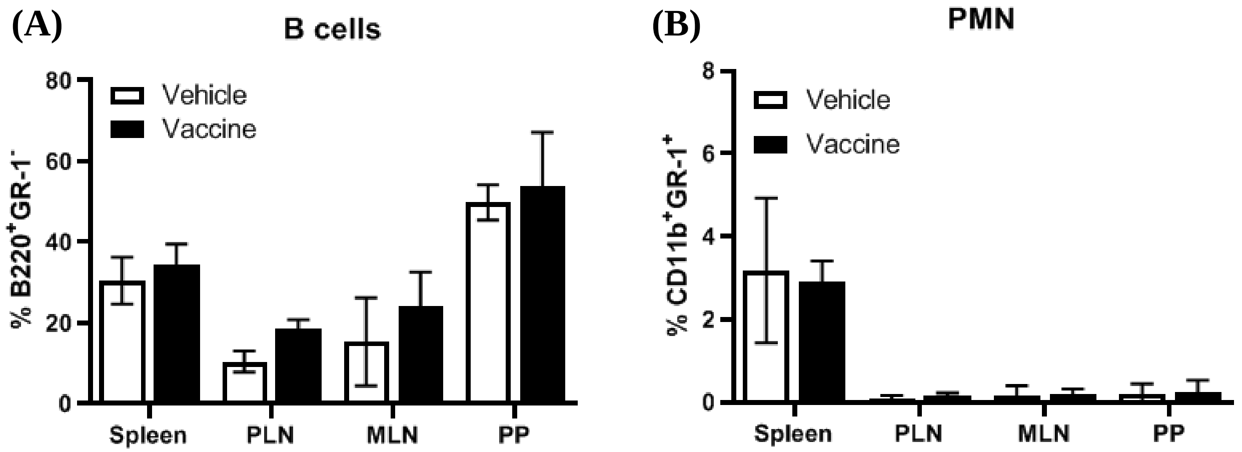

Supplementary Figure 1: A *Salmonella*-based vaccine does not impact B cell and polymorph nuclear macrophage numbers in lymphoid organs. NOD mice were injected with 2.5 µg/mouse of anti-CD3 mAb for 5 days followed by 2 doses of oral combined PPI+TGFβ+IL10 vaccine (n=5). Four weeks post-vaccination, B cells and PMN were analyzed in different organs by FACS analysis. **(A)** Quantification of the percentage of B220^{+}GR-1^{-} (B cells) in total live cells of the spleen, PLN, MLN, and PP. **(B)** Quantification of the percentage of CD11b^{+}GR-1^{+} (PMN) in total live cells of the spleen, PLN, MLN, and PP. The data shown are the average with SD from 2 independent experiments. Statistical analysis using two-way ANOVA shows significance between vaccine and vehicle treated groups (*p<0.01, ***P<0.001, ****p<0, 0001).
